# Low Percentage of Signal Regulatory Protein α/β^+^ Memory B Cells in Blood Predicts Development of Anti-drug Antibodies (ADA) in Adalimumab-Treated Rheumatoid Arthritis Patients

**DOI:** 10.3389/fimmu.2018.02865

**Published:** 2018-12-05

**Authors:** Laura Magill, Marsilio Adriani, Véronique Berthou, Keguan Chen, Aude Gleizes, Salima Hacein-Bey-Abina, Agnes Hincelin-Mery, Xavier Mariette, Marc Pallardy, Sebastian Spindeldreher, Natacha Szely, David A. Isenberg, Jessica J. Manson, Elizabeth C. Jury, Claudia Mauri

**Affiliations:** ^1^Division of Medicine, Centre for Rheumatology, University College London, London, United Kingdom; ^2^Sanofi, Chilly-Mazarin, France; ^3^Clinical Immunology, GlaxoSmithKline, Philadelphia, PA, United States; ^4^INSERM UMR996, Faculté Pharmacie, Université Paris Sud, Châtenay-Malabry, France; ^5^Clinical Immunology Laboratory, AP-HP, Le Kremlin-Bicêtre Hospital, Paris-Sud University Hospitals, Le Kremlin-Bicêtre, France; ^6^UTCBS, CNRS UMR 8258, INSERM U1022, Faculty of Pharmacy, Paris-Descartes-Sorbonne-Cité University, Paris, France; ^7^Centre for Immunology of Viral Infections and Autoimmune Diseases, INSERM UMR1184, AP-HP, Université Paris-Sud, Hôpitaux Universitaires Paris-Sud, Le Krelin-Bicetre, France; ^8^Novartis Pharma AG, Novartis Institutes for BioMedical Research, Basel, Switzerland; ^9^Department of Rheumatology, University College London Hospital, London, United Kingdom

**Keywords:** B cells, rheumatoid arthritis, anti-drug antibodies, immunogenicity, SIRP, anti-TNF, adalimumab, memory B cells

## Abstract

An important goal for personalized treatment is predicting response to a particular therapeutic. A drawback of biological treatment is immunogenicity and the development of antibodies directed against the drug [anti-drug antibodies (ADA)], which are associated with a poorer clinical outcome. Here we set out to identify a predictive biomarker that discriminates rheumatoid arthritis (RA) patients who are more likely to develop ADA in response to adalimumab, a human monoclonal antibody against tumor necrosis factor (TNF)α. By taking advantage of an immune-phenotyping platform, LEGENDScreen™, we measured the expression of 332 cell surface markers on B and T cells in a cross-sectional adalimumab-treated RA patient cohort with a defined ADA response. The analysis revealed seven differentially expressed markers (DEMs) between the ADA^+^ and ADA^−^ patients. Validation of the DEMs in an independent prospective European cohort of adalimumab treated RA patients, revealed a significant and consistent reduced frequency of signal regulatory protein (SIRP)α/β-expressing memory B cells in ADA^+^ vs. ADA^−^ RA patients. We also assessed the predictive value of SIRPα/β expression in a longitudinal RA cohort prior to the initiation of adalimumab treatment. We show that a frequency of < 9.4% of SIRPα/β-expressing memory B cells predicts patients that will develop ADA, and consequentially fail to respond to treatment, with a receiver operating characteristic (ROC) area under the curve (AUC) score of 0.92. Thus, measuring the frequency of SIRPα/β-expressing memory B cells in patients prior to adalimumab treatment may be clinically useful to identify a subgroup of active RA subjects who are going to develop an ADA response and not gain substantial clinical benefit from this treatment.

## Introduction

Rheumatoid arthritis (RA) is a chronic autoimmune disease that manifests as pain, swelling and stiffness of the joints (1), with a 0.5–1% prevalence in the adult population (2). Loss of tolerance drives disease onset and the associated excessive inflammation can lead to destruction of cartilage and bone erosion (1) as well as to systemic complications including cardiovascular disorders, if left untreated (3). Over the last few years the introduction of several biological drugs has significantly improved the treatment of RA, particularly for those patients that fail to control disease with the use of conventional disease modifying anti-rheumatic drugs (cDMARDs). Drugs neutralizing pro-inflammatory cytokines including TNFα (adalimumab, infliximab, and etanercept) and IL-6 (tocilizumab) as well as depleting B cells (rituximab), and blocking T cell function (abatacept), are successfully used in the treatment of RA (4).

A significant percentage of patients with RA receiving biologics will mount an immune response against the drug (immunogenicity), leading to the production of anti-drug antibodies (ADA), often within the first 6 months of therapy (5). This is associated with lower blood drug levels, unresponsiveness to the drug, and a worse clinical outcome (6). Multiple clinical studies have shown that the incidence of ADA differs depending on the biological therapy administered. Immunogenicity occurs in around 33% of adalimumab- and 63% of infliximab-treated RA patients. A lower occurrence is reported after rituximab or tocilizumab treatment with 11% and < 8% of RA patients, respectively, developing ADA (7–15). Circulating ADAs can form immune complexes that increase the clearance of the drug. The majority of ADAs against adalimumab have neutralizing capacity and prevent the drug from interacting with its target (16). Of interest, lower drug levels combined with the presence of ADA at 3 months after treatment initiation, predicted lack of response to adalimumab at 12 months (17). Since not all patients treated with biological therapies develop ADA, the ability to identify a biomarker/s to predict immunogenicity would considerably improve the clinical management of RA.

B cells play a prominent role in RA pathogenesis via the production of rheumatoid factor (RF) and anti-cyclic-citrullinated peptide (anti-CCP) autoantibodies, in addition to antibody-independent roles (18). B cells in the periphery can be defined according to stages of maturation: immature CD24^hi^CD38^hi^; memory CD24^hi^CD38^lo^; and mature CD24^int^CD38^int^B cells. Numerical and functional alterations in B cell subsets have been consistently reported in patients with RA (18). Of interest IgD^+^CD27^+^memory B cells are enriched in the synovium, compared to the peripheral blood, suggesting that the migration of pathogenic memory B cells into inflamed synovium may contribute to disease progression (19). Furthermore, RA patients with active disease have been reported to have a reduced frequency of regulatory B cells, which also exhibit functional impairment (20).

Adalimumab is a fully human monoclonal antibody against TNFα, which is often used as a first line biological therapy for patients, who have failed to respond to treatment with cDMARDs (21). We investigated cell surface markers associated with the development of ADA in RA patients treated with adalimumab using a high-throughput flow cytometry technology (LEGENDScreen™) combined with a systematic analysis framework (SAF). Firstly, an immune-module composed of 7 differentially expressed markers (DEMs) discriminating ADA^+^ vs. ADA^−^ RA patients, was identified in B cell subsets in a cross-sectional cohort of adalimumab-treated RA patients (UK-resident). Validation of the immune-module in an independent prospective European cohort, analyzed 12 months after adalimumab initiation, shows that the frequency of SIRPα/β^+^memory B cells was significantly lower (< 9.4%) in ADA^+^ compared to ADA^−^ RA patients. Moreover, we show that this low frequency of SIRPα/β^+^memory B cells prior to adalimumab treatment (< 9.4%) is able to predict the development of ADA in the same prospective European cohort of RA patients. Thus, we propose that enumeration of memory B cells expressing SIRPα/β prior to initiation of adalimumab could prevent unnecessary treatment of patients that are unlikely to respond to this therapy due to development of ADA.

## Materials and Methods

### Study Design

A UK cross-sectional cohort of 20 adalimumab treated RA patients and a European prospective cohort of 37 RA patients switching to adalimumab treatment were recruited for this study as part of the ABIRISK consortium (Anti-Biopharmaceutical Immunization: prediction and analysis of clinical relevance to minimize the RISK; www.abirisk.eu/). PBMCs and serum samples were obtained and clinical information recorded for time of visit. Peripheral blood was obtained from patients attending the Rheumatology clinic at University College London Hospital, London or at other ABIRISK centers in the France, Netherlands and Italy. All patients matched the definition of RA, as outlined in the ACR-EULAR classification (revised 2010, 1992 ACR original). We collected detailed clinical and laboratory information corresponding to day of sampling, from clinical records where available including: DAS28 score, C-reactive protein (CRP), rheumatoid factor (RF), erythrocyte sedimentation rate (ESR), and anti-cyclic citrullinated peptide antibody (anti-CCP), smoking status, gender, age, and body mass index (BMI). We excluded sero-negative (RF and CCP negative) patients, to reduce the risk of inclusion of patients with spondyloarthropathy, which can sometimes be classified within groups of seronegative RA patients. For the cross-sectional study peripheral blood was taken at a single time point from patients who were currently undergoing or had previously been on adalimumab treatment. Forty-five milliliter (35 ml in France) of blood was collected in Heparin/Sodium Vacutainer blood collection tubes for PBMC isolation (BD) and a further 5 ml serum sample is obtained in serum Vacutainer SST tubes (BD). Patients with RA that were about to start adalimumab treatment, having previously been treated with either a different biological therapy or with cDMARDs, were recruited to the prospective study. These patients were followed longitudinally with collection of serum and PBMC samples at baseline prior to starting adalimumab, and subsequently at 1 and 12 months post commencement of treatment. To minimize batch effect, PMBCs and serum were frozen at each visit to allow simultaneous assessments to be performed for a given individual. PBMCs were isolated using density gradient centrifugation with Ficoll-Paque Plus (GEHealthcare) and frozen at −80°C in autologous plasma with 10% DMSO, and stored in liquid nitrogen. Serum tubes were centrifuged to separate cellular material from the serum and serum aliquots frozen and stored at −80°C. Ethical approval was obtained from the ethics committee of; University College London Hospitals Health Service Trust (14/LO/0506 and 14/SC/1200); CPP, Ile de France VII (13-048), Academic Medical Centre, Amsterdam (METC 2013_304), and Azienda Ospedaliero Univeritaria Careggi (2012/0035P82), and all patients provided written informed consent.

### Anti-drug Antibodies Measurements

Adalimumab ADA were measured using MSD GOLD 96-well Streptavidin SECTOR Plates (L15SA) and a Meso Scale Discovery (MSD) MESO® QuickPlex SQ 120 Instrument. The MSD technology is based on electrochemiluminescence in a bridging format. Adalimumab (Humira®, Abbvie) was independently labeled with either EZ-Link® Sulfo-NHS-LC-Biotin (Thermo scientific 21327) or MSD SULFO-TAG NHS-Ester (Meso Scale Discovery, R91AO) according to the supplier's instructions. Plasma samples diluted at 1/10 (Minimum required dilution) from patients were incubated overnight with biotin-labeled adalimumab and then transferred on GOLD 96-well Streptavidin SECTOR Plates (L15SA) blocked prior with 150 μl of casein in PBS. The plate was then read on a MSD Sector Imager. The negative control was composed of a pool of plasma from 10 negative healthy donors supplied by the French Blood Bank (EFS, Rungis, France). The positive control was a pool of four monoclonal anti-Adalimumab antibodies produced and purified from a human B cell hybridoma by the Immune Regulation Laboratory Institute for Research in Biomedicine (IRB, Bellinzona) in the frame of the ABIRISK program. The sensitivity of the assay was determined by testing eight serial dilutions of the positive control spanning the cut point in three independent assays. The sensitivity was 18 ng/ml for anti-Adalimumab.

### LEGENDScreen™ Immune Phenotyping and Multi-Color Flow Cytometry

PBMCs were phenotyped using LEGENDScreen™ kits, as per the manufacturers instructions. Data was collected on the BD Verse flow cytometer and analyzed using FlowJo 8.7 software (TreeStar).

For multi-color flow cytometry samples were stained with live/dead fixable blue (Invitrogen), for 20 min at RT and antibodies for flow cytometry, for 30 min at 4°C as follows; CD19 BV785 (HIB19), CD24 PECy7 (ML5) (BD), CD38 BV605 (HIT2, BV605), to define B cells and subsets, and combinations of the following; SIRPα/β APC (SE5A5), CD324 APC-Fire750 (67A4), CD1c BV421 (L161), CD127 BV510 (AO19D5), CD1a PE-Dazzle594 (HI149), CD167a PE (S1D6), CD138 APC (DL-101) (all BioLegend unless stated otherwise). Appropriate FMO controls were used, and samples were fixed before acquiring on the flow cytometer. Data was collected on the BD LSR2 flow cytometer (BD), and analyzed using FlowJo version 8.7 or 10.5.

### Statistical Analysis

Statistical analyses were performed using Prism version 6 (Graphpad) and JMP (SAS) version 12. *P* < 0.05 were considered as significant. Heatmaps were generated using Multiple Experiment Viewer_4_8 (MeV_4_8) (TM4). DEMs were determined using a two-tailed *t*-test or one-way ANOVA where appropriate. Fold change was determined by calculating the difference between groups as a ratio. Graphs show individual data points, with mean and ±SEM. Outliers were removed in the validation analysis using the ROUT method (Robust regression and Outlier removal) in Prism (Q = 1%), where stated. Principle component analysis (PCA) was performed using JMP. Receiver operating characteristic (ROC) curves were generated and area under the curve (AUC) calculated in JMP.

## Results

### LEGENDScreen™ Analysis Identifies an Immune-Module Associated With ADA in RA Patients Treated With Adalimumab

**Figure 1:**
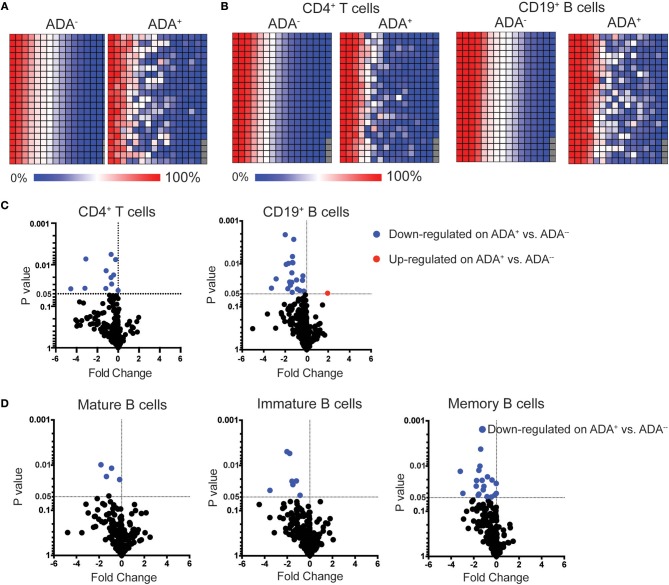
LEGENDScreen™ analysis of adalimumab treated RA patients (cross-sectional cohort) identifies differences between ADA^+^ and ADA^−^ individuals. PBMCs from patients treated with adalimumab defined as ADA^+^ (*n* = 10) or ADA^−^ (*n* = 10) were stained with LEGENDScreen™ for 332 cell surface markers, in addition to antibodies against CD19, CD4 and CD24 and CD38. Heatmaps showing average frequency expression of each LEGENDScreen™ marker for each sample group (ADA^+^ and ADA^−^) for PBMCs **(A)**, and CD4^+^T cells and CD19^+^B cells **(B)**. Markers are ranked according to expression in ADA^−^ patients. Volcano plot showing fold-change of frequency expression between patient groups (ADA^−^/ADA^+^) (Log_2_) and *p*-value (*t*-test) (Log_10_), no markers passed the Holm-Sidak *post-hoc* test. Blue circle: significantly down-regulated markers; red circle significantly up-regulated markers, in ADA^−^ vs. ADA^+^; **(C)** for CD4^+^T cells and CD19^+^B cells, and **(D)** mature (CD24^int^CD38^int^), immature (CD24^hi^CD38^hi^), and memory (CD24^hi^CD38^lo^) B cells.

To identify biomarker/s associated with ADA response to adalimumab we examined the surface immune-signature of PMBCs isolated from 10 ADA^−^ to 10 ADA^+^ RA patients treated with adalimumab for a minimum of 12 months (Table 1). PBMCs were stained with fluorescently-conjugated antibodies identifying CD4^+^T cells, CD19^+^B cells, immature, mature, and memory B cell subsets in addition to the 332 cell surface markers included in the LEGENDScreen™ panel (Figure S1). LEGENDScreen™ inter- and intra-assay reproducibility was validated in an independent study (22). The expression-pattern of surface markers on PBMCs appeared to be distinct between ADA^−^ and ADA^+^ RA patients (Figure 1A). The variation in marker expression is also present following analysis of CD19^+^B cells and CD4^+^T cells (Figure 1B), with a greater difference observed in CD19^+^B cells than in CD4^+^T cells. As many of the markers assessed by the LEGENDScreen™ platform were either not expressed or were expressed at a very low level, to increase the statistical power of future analyses, markers with < 5% expression on all samples tested were excluded from this study. Inclusion of this criteria resulted in the removal of 74 B and 134 T cell markers. Expression of the remaining markers was compared between ADA^+^ and ADA^−^ RA patients using multiple *t*-test analysis. CD19^+^B cells showed highest number of DEMs (ADA^−^ vs. ADA^+^) (*n* = 22) compared to CD4^+^T cells (*n* = 11) (*p* < 0.05, multiple *t*-test) (Figure 1C). These results together with the numerical and functional imbalance in CD19^+^B cells associated with the pathogenesis of RA (18–20), prompted us to focus on DEMs expressed by B cell subsets. Respectively, four, seven, and 19 DEMs were identified on mature, immature, and memory B cells between ADA^+^ and ADA^−^ RA patients (Figure 1D, Table S1). Of note, all these DEMs are down-regulated in ADA^+^ RA patients.

**Table 1:** Patient demographic and disease characteristics for LEGENDScreen™ discovery cohort (UK based).

	**Healthy Controls**	**RA (adalimumab ADA^−^)**	**RA (adalimumab ADA^+^)**	**DMARDs treated RA (biologic naïve)**
*n*	18	10	10	10
Sex, female *n* (%)	15 (79)	7 (70)	9 (90)	9 (90)
Age (years), mean (*SD*)	35.2 (10.7)	62.4 (14.8)	53.4 (10.5)	45.6 (13.5)
DAS28 (*SD*)	–	3.05 (1.06)	3.63 (1.69)	3.95 (1.76)
Seropositive (RF+/CCP+) (%)	–	100	90	100
CRP mg/l (*SD*)	–	14.07 (21.54)	5.64 (6.20)	6.78 (4.75)
**CURRENT TREATMENT**				
DMARDs only (*n*)	–	3^*^	1^*^	10
Adalimumab (*n*)	–	5	8	–
Etanercept (*n*)	–	1^*^	–	–
Tocliziumab (*n*)	–	1^*^	1^*^	–
**CONCOMITANT DMARD TREATMENT**				
MTX use, *n* (%)	–	7	5	5
Average MTX dose, mg/week, mean (*SD*)	–	15.7 (3.6)	18 (51.1)	19 (2.0)
Prednisolone use, *n* (%)	–	1	2	2
Hydroxychloroquine use, *n* (%)	–	1	2	6
Sulfasalazine use, *n* (%)	–	1	1	6

*In the discovery cohort there are no statistical differences between ADA^+^ and ADA^−^ RA patient groups (t-test, p < 0.05 was considered as significant), nor between ADA^+^ and ADA^−^ and DMARDs treated (ANOVA), expect for age between ADA^−^ and DMARDs*.

* *At point of sampling patients receiving a non-adalimumab treatment as indicated (Current Treatment) but have previously been treated with adalimumab and tested for ADA against adalimumab*.

*Values in the table represent mean ± standard deviation (SD), or number of patients (n) with proportion of total (%) where indicated*.

*Adalimumab dose 40 mg every 2 weeks, tocilizumab dose 4 mg/kg every 4 weeks, etanercept does 25 mg twice a week*.

**Figure 2:**
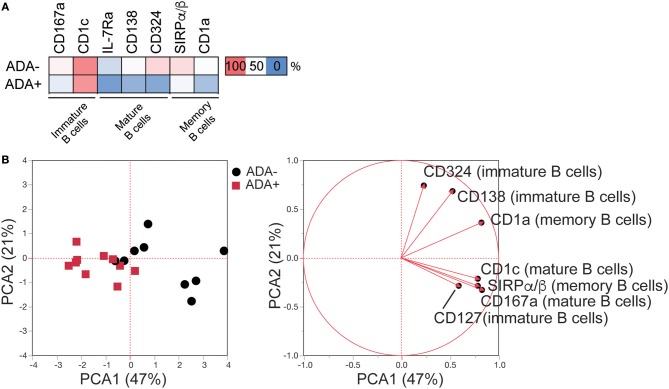
LEGENDScreen™ analysis of adalimumab treated RA patients (cross-sectional cohort) identifies an immune-module associated with ADA. LEGENDScreen™ analysis as Figure 1. **(A)** Heatmap showing mean frequency of differentially expressed markers (DEMs) (*p*-value defined in Table S1), between patient groups on B cell subsets, following exclusion of markers associated with DAS28 and “treatment effect” (see Figure S2 for details). **(B)** PCA of frequency of expression of the DEMs on the 20 adalimumab treated RA patients (ADA^−^ black circles, ADA^+^ red squares) with contribution of each marker to the principal components denoted by length and direction of the corresponding red arrow.

Next, a systematic framework analysis (SFA) was used to distinguish between DEMs associated with the presence of ADA as opposed to disease severity and/or the effect of adalimumab therapy (Figure S2). DEMs expressed on memory B cells that correlated significantly with Disease Activity Score-28 (DAS28) (*n* = 1) (Pearson correlation) were excluded from our study, as these were considered to be due to RA-related inflammation (Table S2). None of the DEMs identified on mature and immature B cells correlated with DAS28. To account for treatment effect, we compared the expression of the DEMs in ADA^−^, ADA^+^, and cDMARD treated RA patients (RA-D). Using one-way ANOVA analysis, we excluded DEMs that were significantly different between ADA^−^ and RA-D, but not between ADA^+^ and RA-D, as these were considered to be treatment related (Figure S3). Furthermore, we removed any DEMs that no longer showed significance following ANOVA analysis between ADA^−^ and ADA^+^ samples. The application of the SFA, followed by the application of an unbiased principal component analysis (PCA), demonstrates that 7 remaining DEMs, specifically CD167a and CD1c expressed on mature; IL-7Rα, CD138, and CD324 on immature and SIRPα/β and CD1a on memory B cells (expression shown in heatmap; Figure 2A) have sufficient statistical power to discriminate between ADA^+^ and ADA^−^ RA patients (Figure 2B); henceforth defined as the “module.”

### A Frequency of < 9.4% of SIRPα/β^+^Memory B Cells Constitutes a Risk Factor for ADA Development

**Figure 3:**
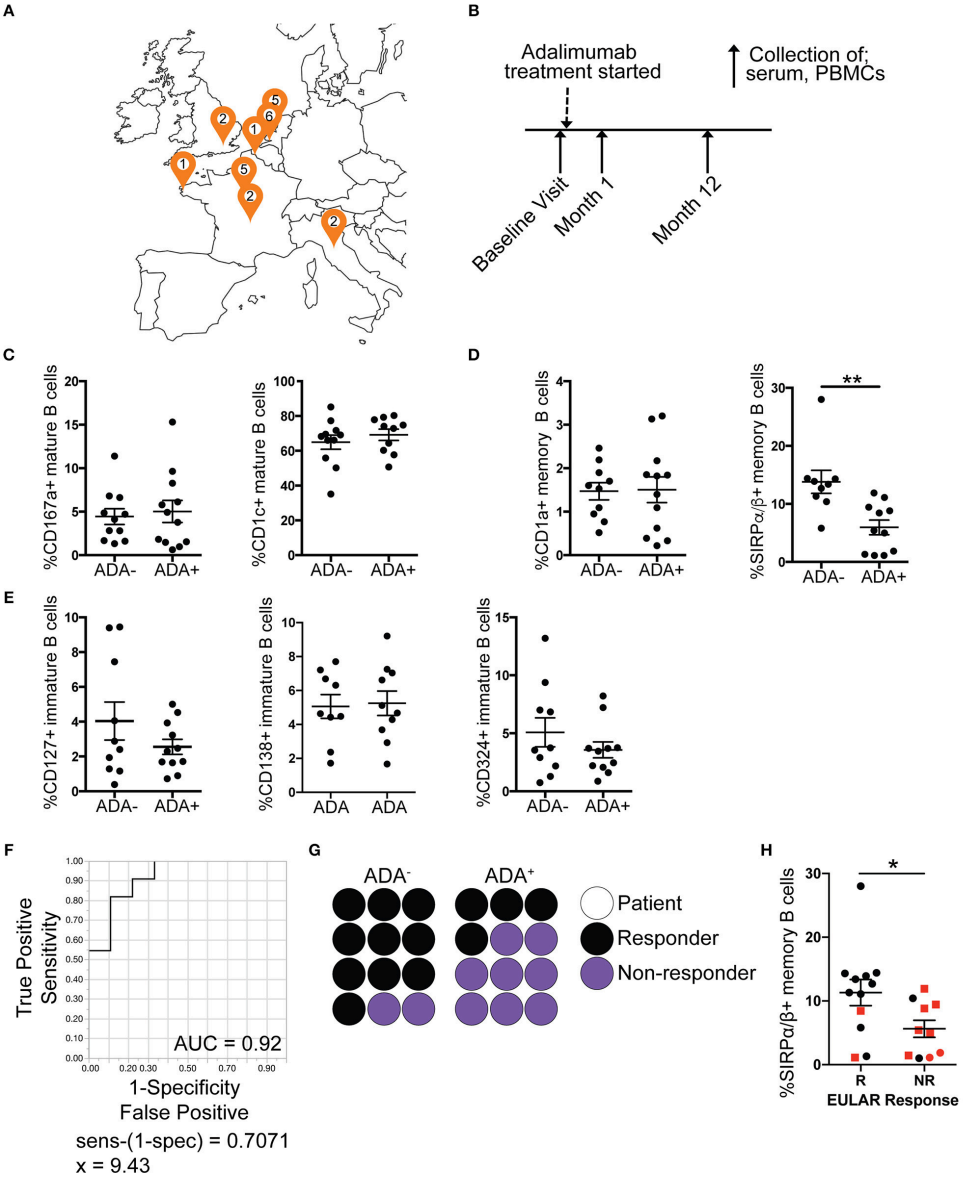
Reduced frequency of SIRPα/β^+^ memory B cells is validated in ADA^+^ adalimumab treated RA patients. PBMCs and serum samples were collected longitudinally (baseline, month 1 and 12 following start of treatment), from RA patients starting adalimumab treatment across Europe. For each visit ADA level was measured by Meso Scale Discovery (MSD) technology. Month 12 PBMCs were stained for flow cytometry, for the module (7 markers), and the frequencies of cells expressing the markers analyzed (*n* = 12 ADA^−^, *n* = 12 ADA^+^). Outliers removed using robust regression and outlier removal [ROUT (Q = 1%)]. **(A)** Location of recruitment sites, and number of patients recruited following exclusions (Figure S4). **(B)** Timeline of sample collection. Frequency of expression of DEMs on **(C)** mature, **(D)** memory and **(E)** immature B cells from the prospective cohort; scatter plots showing mean ±SEM. *T*-test analysis, ^**^p ≤ 0.01. **(F)** Receiver operating characteristic (ROC) curve for %SIRPα/β^+^memory B cells, AUC reported in figure. **(G)** Dot plot representing proportion of ADA^−^ and ADA^+^ patients that are responders (black) or non-responders (purple); each circle represents one prospective patient. **(H)** Scatter plot with mean and ±SEM of %SIRPα/β^+^memory B cells for non-responder (NR) and responder (R) patients to adalimumab according to EULAR classification, red squares represent ADA^+^ patients and black circles ADA^−^ patients. *T*-test analysis, ^*^p ≤ 0.05.

While previous studies have shown some molecular association with the development of ADA in RA patients (23, 24), currently there are no clinically accepted predictive biomarkers for ADA development in anti-TNFα treatment. We validated the B cell subset ADA-associated “module” in an independent prospective European RA cohort (*n* = 37) (map of recruitment is shown in Figure 3A), which was designed to assess immunogenicity development following initiation of adalimumab treatment (none of the patients included in this study had been previously treated with adalimumab) (Table 2). Purified PBMCs and serum were collected at three time points: baseline prior to commencement of treatment, 1 month and 12 months after treatment initiation (Figure 3B), and ADA was measured at each time point. Out of the 37 patients recruited, 5 seronegative (RF and CCP negative) and 2 of unknown serological status were excluded from this study, to reduce the risk of inclusion of patients with spondyloarthropathy (Figure S4). A further 6 patients, which tested positive at baseline (*n* = 5) or had transient ADA expression (*n* = 1) were excluded.

**Table 2:** Patient demographic and disease characteristics for independent prospective validation cohort (UK, France, Italy, Netherlands).

	**Healthy Controls**	**RA (adalimumab ADA^−^)**	**RA (adalimumab ADA^+^)**
*n*	31	12	12
Sex, female *n* (%)	24 (68)	8 (67)	10 (83)
Age (years), mean (*SD*)	27 (10.7)	59 (15.7)	45 (12.6)
DAS28 (*SD*)	–	2.45 (1.2)	3.49 (1.2)
Seropositive (RF+/CCP+) *n* (%)	–	12 (100)	12 (100)
CRP mg/l (*SD*)	–	7.5 (11.6)	9.2 (9.1)
**LOCATION**			
UK		1	1
France		6	3
Italy		1	1
Netherlands		4	7
**CONCOMITANT DMARD TREATMENT**			
MTX use, *n* (%)		10 (83.3%)	8 (66.7%)
Average MTX dose, mg/week, mean (*SD*)		18.25 (5.3)	15.63 (6.6)
Prednisolone use, *n* (%)		1 (8.3%)	3 (25%)
Prednisone use, *n* (%)		3 (25%)	3 (25%)
Hydroxychloroquine use, *n* (%)		3 (25%)	0 (0%)
Leflunomide use, *n* (%)		0 (0%)	3 (25%)
Sulfasalazine use, *n* (%)		2 (17%)	0 (0%)

*CRP and DAS28 are not statistically different; age is statistically different (p = 0.04) between ADA^+^ and ADA^−^ RA cohorts*.

*Values in the table represent mean ± standard deviation (SD), or number of patients (n) with proportion of total (%) where indicated*.

*Month 12 (M12)*.

*All patients received 40 mg adalimumab every other week*.

Month 12 PBMCs from the prospective cohort were stained for CD19, CD24, CD38, and the 7 DEMs as shown in Figure 2A. Analysis showed that the frequency of SIRPα/β^+^memory B cells was consistently reduced in ADA^+^ compared to ADA^−^ RA patients at the 1-year follow-up [*t*-test following removal of outliers (ROUT Q = 1%) (25)]. None of the other module markers were confirmed in this analysis (Figures 3C–E). To test the ability of SIRPα/β^+^memory B cells to distinguish patients as ADA^+^ or ADA^−^ we generated a receiver operating characteristic (ROC) curve, plotting sensitivity against specificity for different SIRPα/β values observed in our validation cohort at month 12 (Figure 3F). An area under the curve (AUC) value of 0.92 was calculated, indicating that the frequency of SIRPα/β^+^memory B cells is highly accurate at defining ADA positivity. A cut-off value of 9.4% SIRPα/β^+^memory B cells was determined using the calculation of sensitivity-(1-specificity), with individuals expressing < 9.4% deemed to be ADA^+^.

To further confirm that changes in SIRPα/β^+^memory B cell frequencies were associated with ADA and not to any of the other clinical or demographic parameters implicated in RA (DAS28, CRP, ESR, age, BMI, ADA titer), a correlation analysis of these variables was performed. None of the parameters analyzed correlated significantly with the percentage of SIRPα/β^+^memory B cells (Figure S5A). Furthermore, there were no significant differences in the percentage of SIRPα/β^+^memory B cells between patients stratified according to concomitant treatment with methotrexate (MTX), gender or smoking status (Figures S5B–D).

Of interest, the development of ADA in the prospective cohort was associated with non-response or partial response to adalimumab, according to the EULAR classification (67%) (Figure 3G). The frequency of SIRPα/β^+^memory B cells was significantly decreased in non-responders compared to responder patients (Figure 3H). However, patient response to treatment was independent from the quantities of ADA in circulation, since ADA titer did not correlate with DAS28 (Figure S5E). Furthermore, the frequency of SIRPα/β^+^memory B cells does not predict response to treatment in patients treated with cDMARDs only, suggesting that frequency of SIRPα/β^+^memory B cells is specific to ADA associated failure of treatment (Figure S6). We therefore hypothesize that the presence of < 9.4% SIRPα/β^+^memory B cells in RA patients prior to initiation of adalimumab treatment could be used as biomarker to predict ADA development in these patients.

### Frequency of SIRPα/β^+^Memory B Cells as a Predictor of ADA

**Figure 4:**
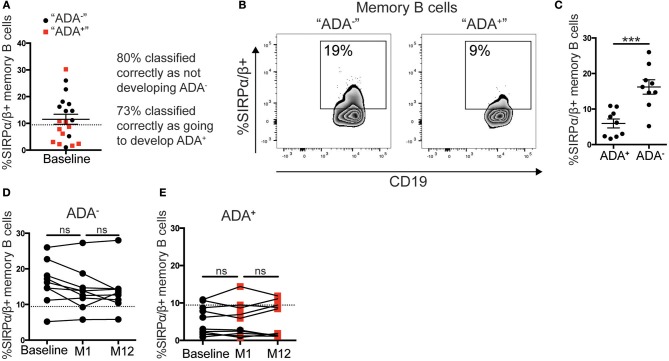
Low %SIRPα/β^+^memory B cells predict ADA development in adalimumab treated RA patients. PBMCs from the cohort described in Figure 3 taken at baseline, month 1 (M1) and 12 (M12) visits (*n* = 21), were stained with SIRPα/β-APC for flow cytometry (*n* = 11 ADA^+^ *n* = 10 ADA^−^). **(A)** RA patients prior to commencing adalimumab (baseline, from prospective cohort) were stratified according to the frequency of SIRPα/β^+^memory B cells. Black circle and red square correspond to individuals, respectively, that do not “ADA^−^” or do “ADA^+^” develop ADA, after unblinding. Dotted line is the threshold value from M12 ROC curve analysis. **(B)** Representative flow cytometry plots showing %SIRPα/β^+^memory B cells in an “ADA^−^” and an “ADA^+^” individuals. **(C)** Scatter plot showing mean ± SEM of %SIRPα/β^+^memory B cells of patients at baseline, sub-divided by ADA development by M12, *t*-test analysis, following a robust regression and outlier removal [ROUT (Q = 1%)], identifying two outliers, ^***^p ≤ 0.0001. **(D)** Graph showing longitudinally the %SIRPα/β^+^memory B cells at baseline, M1 and M12 for ADA^−^ patients stratified as **(C)**, dotted line as 4A, one-way ANOVA. **(E)** %SIRPα/β^+^memory B cells for ADA^+^ patients; red square shows positive ADA detection, black circle shows negative ADA detection, at sampling.

We envisaged two possible scenarios for the change in the frequency of SIRPα/β^+^memory B cells: (i) all patients prior to adalimumab treatment express a similar frequency of SIRPα/β^+^memory B cells, and the frequency is down regulated concomitantly to the development of ADA; (ii) patients that will go on to develop ADA have fewer SIRPα/β^+^memory B cells at baseline compared to the ADA^−^ patients. Using the cut-off value determined by ROC-curve analysis, described in Figure 3F, 9 out of 20 patients assessed at baseline showed < 9.4% of SIRPα/β^+^memory B cells in circulation. Strikingly, 73% of patients with SIRPα/β^+^memory B cell frequencies below the cut-off value became ADA^+^ after 12 months of adalimumab therapy whilst 80% of patients with SIRPα/β^+^memory B cell frequencies above the cut-off value remained ADA^−^ (Figure 4A). Representative expression of SIRPα/β on memory B cells in ADA^−^ vs. ADA^+^ RA patients is shown in Figure 4B. To confirm the predictive value of reduced SIRPα/β^+^ memory B cells frequency, baseline samples were separated according to future development of ADA by month 12. Patients that will develop ADA showed significantly lower frequencies of SIRPα/β^+^memory B cells compared to patients that do not develop ADA (*p* = 0.0002) (Figure 4C). Longitudinal analysis revealed no changes in the frequency of SIRPα/β^+^ memory B cells between visits in both ADA^−^ and ADA^+^ patients (Figures 4D,E). Furthermore, analysis of the month 1 visit revealed that the majority (80%) of patients that have developed ADA by month 12 already have detectable ADA by 1 month following start of treatment (Figure 4E).

## Discussion

Biological therapies are routinely used to treat autoimmune conditions such as RA and have significantly improved the management of these diseases. Despite their success, some patients develop immune reactions against these therapies, and develop ADA, which often leads to treatment failure (10, 17, 26–28). Different drugs possess different degrees of immunogenicity, for example adalimumab, a whole mAb, is more immunogenic than etanercept, a fusion protein that contains only the Fc portion of an antibody (26, 29). While to a certain extent, increasing drug dosage has been shown to alleviate the side effects associated with the presence of ADA (30), there is a need to be able to predict which individuals will develop ADA. Here we aimed, using extensive immune-phenotyping, to identify a predictive biomarker associated with ADA. By using a UK cross-sectional cohort of adalimumab-treated RA patients, followed by validation in a European prospective cohort, we have identified that a reduced frequency of SIRPα/β^+^memory B cells prior to adalimumab treatment, compared to ADA^−^ RA patients, allows the prediction ADA development in RA patients. In addition, we show that the lower frequency of SIRPα/β^+^memory B cells, remains relatively stable following treatment with adalimumab. In general, one of the major pitfalls in this type of study is the lack of reproducibility of a biomarker. This is often due to the absence of validation in independent cohorts (31–33). We mitigated this problem by validating our results in an independent cohort that included patients of mixed ethnicity. It would be important to investigate whether the presence of < 9.4% of SIRPα/β^+^memory B cells also predicts ADA formation in patients with other autoimmune diseases treated with biologics [e.g., inflammatory bowel disease (IBD) treated with adalimumab] or in RA patients treated with other biologics (e.g., infliximab). This would allow us to establish whether the development of ADA is a feature of autoimmunity or is disease/treatment specific.

Studies looking at the efficacy of the biosimilar to the original drug have shown that the biosimilars have comparable efficacy (34). It has been demonstrated that in patients who have developed ADA against infliximab, the ADA will cross-react with the biosimilar, as shown for Remsima in IBD (35) and Inflectra in RA (36). On this basis we would expect the frequency of SIRPα/β^+^memory B cells to also be reduced in patients that lack response to/have ADA against adalimumab biosimilars, however this will be subject to future investigation.

It is tempting to propose that the measurement of the frequency of SIRPα/β^+^memory B cells could also be used as a surrogate marker for ADA in the clinic for patients already being treated with adalimumab. This could be done in combination with or as an alternative and more practical method to those already existing (37). The ability to detect ADA could help to inform clinicians as to when switching treatment is most needed. Overall, the ability to detect ADA in patients already on treatment would potentially deliver precision medicine to this heterogeneous disease and increase the efficiency of clinical decision-making.

There is currently no functional data showing the role of SIRP in B cells. SIRPα has been shown to have a possible role in auto-immunity having been found to be a risk loci in individuals with Type 1 Diabetes (38), and Crohn's patients have increased SIRPα/β^+^CD11C^+^DCs in the mesenteric lymph nodes and inflamed intestinal mucosa (39). SIRP alpha and beta are members of the Signaling Inhibitory Receptor Protein (SIRP) family, and are membrane expressed proteins. Predominantly found on myeloid cells, they act to mediate cell to cell interactions by regulating the type and strength of the signal (40). The major ligand for SIRPα is CD47, which is ubiquitously expressed and has a role in apoptosis, proliferation, adhesion and migration (41), while SIRPβ has no known ligand. It is interesting to note that not all individuals with low frequency of SIRPα/β^+^memory B cells develop ADA, suggesting the existence of specific risk factors, genetic or environmental, that predisposes some individuals to develop ADA.

In this study we have demonstrated the value of measuring the frequency of SIRPα/β^+^memory B cells for the prediction of RA patients at risk of developing ADA against adalimumab. However, one limitation of our study is the sample sizes available due to the low frequency of ADA^+^ patients. Therefore, several outstanding questions remain unanswered and will need to be addressed in a larger cohort of patients. For example, it would be interesting to address whether SIRPα/β is also a genetic risk loci associated with ADA development in patients with RA, and whether the low frequency of SIRPα/β^+^memory B cells in RA patients prior to adalimumab treatment is exclusively a biomarker for ADA response or whether the reduction of this population is also mechanistically implicated in the development of an ADA response. In addition, it is possible that age and other disease specific factors such as previous and concomitant treatments, and disease duration, may also have an effect on ADA development. Of note, our data showing that there is no significant difference in the frequency of SIRPα/β^+^memory B cells between RA patients that are responders vs. non-responders to cDMARD treatment supports that the frequency of SIRPα/β^+^memory B cells is related to an ADA-specific lack of response (Figure S5).

In conclusion we have identified a predictive marker present prior to adalimumab treatment that it is associated with the development of antibodies against adalimumab in patients with RA; specifically, a frequency of < 9.4% of SIRPα/β^+^memory B cells. To our knowledge this is the first extensive immune phenotyping analysis of B cells in a longitudinal cohort of RA patients treated with adalimumab. We envisage that this can be used as part of a toolkit combining other biomarkers, and genetic and other risk factors, that will allow for more personalized treatment.

## Author Contributions

LM, CM, EJ, and JM: study concept, design, and interpretation of data; LM: acquisition and analysis of data; NS and KC: ADA testing; AG, AH-M, SH-B-A, and VB: project administration; LM and CM: drafting of manuscript. All authors made critical revisions of the manuscript for important intellectual content.

### Conflict of Interest Statement

VB and AH-M were employed by Sanofi. KC was employed by GlaxoSmithKline. SS was employed by Novartis. The remaining authors declare that the research was conducted in the absence of any commercial or financial relationships that could be construed as a potential conflict of interest.
