# Supplementary material for: Low Percentage of Signal Regulatory Protein α/β+ Memory B Cells in Blood Predicts Development of Anti-drug Antibodies (ADA) in Adalimumab-Treated Rheumatoid Arthritis Patients
Source: Front Immunol. 2018 Dec 5;9:2865. doi: 10.3389/fimmu.2018.02865 (PMC6290031; doi:10.3389/fimmu.2018.02865)
Supplement: Supplementary file 1 [file Data_Sheet_1.docx]

**SUPPLEMENTARY TABLES AND FIGURES**

**Supplementary Table 1.** LEGENDScreen^TM^ results; differentially expressed markers (DEMs) (*t*-test, p<0.005) on B cells (total) and mature, immature and memory B cell subsets.

|  |  |  |  |  |
| --- | --- | --- | --- | --- |
| **B cells (total)** | | | | |
|  | **P value** | **Mean ADA^-^** | **Mean ADA^+^** | **Fold change** |
| CD158f | 0.002 | 14.4 | 3.6 | 4.0 |
| Notch 4 | 0.003 | 10.3 | 4.5 | 2.3 |
| CD105 | 0.007 | 50.6 | 20.3 | 2.5 |
| CD158d | 0.009 | 16.6 | 6.7 | 2.5 |
| CD1a | 0.009 | 7.6 | 3.1 | 2.4 |
| CD138 | 0.010 | 13.6 | 4.2 | 3.2 |
| CD226 | 0.010 | 25.4 | 6.8 | 3.7 |
| DR3 | 0.016 | 17.5 | 6.7 | 2.6 |
| CD1c | 0.019 | 86.6 | 67.9 | 1.3 |
| CD111 | 0.022 | 39.6 | 5.5 | 7.2 |
| CD338 | 0.024 | 10.3 | 5.4 | 1.9 |
| CD275 | 0.024 | 80.5 | 61.7 | 1.3 |
| CD87 | 0.026 | 33.0 | 11.0 | 3.0 |
| CD172a | 0.027 | 22.1 | 8.5 | 2.6 |
| Nectin-2 | 0.031 | 31.5 | 11.1 | 2.8 |
| CD324 | 0.037 | 35.4 | 3.7 | 9.7 |
| CD276 | 0.038 | 22.1 | 6.5 | 3.4 |
| CD167a | 0.039 | 21.1 | 11.6 | 1.8 |
| CD262 | 0.042 | 18.5 | 11.8 | 1.6 |
| CD165 | 0.043 | 81.2 | 67.2 | 1.2 |
| CD178 | 0.046 | 6.8 | 2.7 | 2.5 |
| FcRL6 | 0.048 | 3.4 | 13.0 | 0.3 |
|  |  |  |  |  |
|  |  |  |  |  |
| **Mature B cells** | | | | |
|  | **P value** | **Mean ADA^-^** | **Mean ADA^+^** | **Fold change** |
| CD158f | 0.010 | 7.3 | 2.0 | 3.6 |
| CD167a | 0.012 | 17.5 | 9.4 | 1.9 |
| CD138 | 0.018 | 6.9 | 2.7 | 2.5 |
| CD1c | 0.021 | 82.8 | 73.2 | 1.1 |
|  |  |  |  |  |
| **Immature B cells** | | | | |
|  | **P value** | **Mean ADA^-^** | **Mean ADA^+^** | **Fold change** |
| IL-7Ra | 0.005 | 7.8 | 1.9 | 4.1 |
| CD138 | 0.005 | 13.3 | 3.8 | 3.4 |
| DR3 | 0.022 | 16.8 | 7.4 | 2.3 |
| CD87 | 0.022 | 32.9 | 11.0 | 3.0 |
| CD158f | 0.027 | 15.5 | 5.5 | 2.8 |
| CD324 | 0.036 | 34.5 | 3.0 | 11.6 |
| CD105 | 0.046 | 49.5 | 27.2 | 1.8 |
|  |  |  |  |  |
| **Memory B cells** | | | | |
|  | **P value** | **Mean ADA^-^** | **Mean ADA^+^** | **Fold change** |
| CD158d | 0.004 | 24.6 | 9.4 | 2.6 |
| CD87 | 0.010 | 37.8 | 13.8 | 2.7 |
| CD172a | 0.012 | 30.2 | 10.3 | 2.9 |
| CD111 | 0.013 | 42.8 | 4.7 | 9.1 |
| CD167a | 0.017 | 24.9 | 14.4 | 1.7 |
| CD275 | 0.020 | 63.4 | 48.6 | 1.3 |
| CD226 | 0.021 | 31.6 | 13.2 | 2.4 |
| CD138 | 0.021 | 18.4 | 5.4 | 3.4 |
| CD59 | 0.024 | 99.7 | 98.8 | 1.0 |
| CD158f | 0.028 | 19.3 | 5.6 | 3.4 |
| CD1a | 0.028 | 11.3 | 5.3 | 2.2 |
| Notch 4 | 0.034 | 13.0 | 5.8 | 2.2 |
| CD324 | 0.040 | 35.6 | 4.6 | 7.8 |
| CD63 | 0.040 | 99.6 | 98.8 | 1.0 |
| CD109 | 0.042 | 17.3 | 5.9 | 2.9 |
| CD1c | 0.044 | 89.4 | 80.9 | 1.1 |
| CD178 | 0.044 | 10.7 | 3.5 | 3.0 |
| CD338 | 0.048 | 17.4 | 9.8 | 1.8 |
| CD97 | 0.048 | 74.1 | 54.2 | 1.4 |

**Supplementary Table 2.** LEGENDScreen^TM^ results; markers significantly correlated with DAS28, on total B cells and B cell subsets (p>0.05).

| **B cells** | | |
| --- | --- | --- |
|  | **r** | **P value** |
| BTLA | -0.521 | 0.006 |
| CD102 | -0.432 | 0.019 |
| CD11a | -0.544 | 0.002 |
| CD11c | 0.470 | 0.013 |
| CD134 | 0.497 | 0.006 |
| CD135 | 0.398 | 0.033 |
| CD152 | 0.404 | 0.033 |
| ADAM10 | -0.523 | 0.004 |
| CD180 | -0.418 | 0.024 |
| CD184 | -0.444 | 0.016 |
| CD196 | -0.434 | 0.019 |
| CD200 | -0.460 | 0.012 |
| CD22 | -0.416 | 0.025 |
| CD229 | 0.401 | 0.034 |
| CD231 | -0.525 | 0.003 |
| CD245 | -0.450 | 0.014 |
| CD252 | 0.381 | 0.046 |
| CD26 | 0.382 | 0.041 |
| CD267 | 0.425 | 0.022 |
| CD268 | -0.373 | 0.046 |
| CD270 | -0.443 | 0.018 |
| CD275 | -0.410 | 0.027 |
| CD319 | 0.539 | 0.003 |
| CD35 | -0.415 | 0.025 |
| CD45RA | -0.410 | 0.027 |
| CD47 | -0.529 | 0.003 |
| CD48 | -0.484 | 0.008 |
| CD53 | -0.436 | 0.018 |
| CD55 | -0.380 | 0.042 |
| CD74 | -0.413 | 0.026 |
| CD99 | -0.387 | 0.038 |
| HLA-A2 | 0.413 | 0.036 |
| HLA-DQ | -0.425 | 0.024 |
| HLA-DR | -0.419 | 0.027 |
| IgD | -0.412 | 0.026 |
|  |  |  |
| **Mature B cells** | | |
|  | **r** | **P value** |
| CD102 | -0.505 | 0.005 |
| CD11a | -0.516 | 0.004 |
| CD11c | 0.458 | 0.016 |
| CD134 | 0.499 | 0.006 |
| CD152 | 0.415 | 0.028 |
| ADAM10 | -0.443 | 0.016 |
| CD166 | -0.387 | 0.038 |
| CD196 | -0.383 | 0.040 |
| CD229 | 0.425 | 0.024 |
| CD231 | -0.519 | 0.004 |
| CD24 | -0.486 | 0.008 |
| CD245 | -0.434 | 0.019 |
| CD270 | -0.432 | 0.022 |
| CD319 | 0.529 | 0.004 |
| CD44 | -0.480 | 0.008 |
| CD47 | -0.500 | 0.006 |
| CD48 | -0.443 | 0.016 |
| CD55 | -0.375 | 0.045 |
| CD99 | -0.411 | 0.030 |
| HLA-A2 | 0.425 | 0.031 |
|  |  |  |
| **Immature B cells** | | |
|  | **r** | **P value** |
| β2 microglobulin | 0.427 | 0.029 |
| BTLA | -0.436 | 0.023 |
| CD11a | -0.418 | 0.024 |
| CD11c | 0.422 | 0.028 |
| CD134 | 0.408 | 0.028 |
| CD148 | -0.447 | 0.015 |
| ADAM10 | -0.405 | 0.030 |
| CD158d | -0.375 | 0.049 |
| CD184 | -0.510 | 0.006 |
| CD200 | -0.424 | 0.022 |
| CD209 | 0.376 | 0.044 |
| CD215 | 0.374 | 0.050 |
| CD229 | 0.448 | 0.017 |
| CD231 | -0.488 | 0.007 |
| CD252 | 0.435 | 0.021 |
| CD275 | -0.389 | 0.037 |
| CD31 | -0.424 | 0.022 |
| CD319 | 0.602 | 0.001 |
| CD360 | -0.385 | 0.047 |
| CD44 | -0.545 | 0.002 |
| CD48 | -0.417 | 0.027 |
| CD49d | -0.403 | 0.034 |
| CD53 | -0.382 | 0.041 |
| CD55 | -0.428 | 0.023 |
| CD6 | 0.375 | 0.045 |
| CD84 | -0.372 | 0.047 |
| CD86 | 0.446 | 0.017 |
| CD9 | -0.412 | 0.027 |
| Ig light chain λ | -0.435 | 0.024 |
| IgD | -0.399 | 0.035 |
| IL-28RA | 0.602 | 0.001 |
| MICA-MICB | 0.381 | 0.042 |
|  |  |  |
| **Memory B cells** | | |
|  | **r** | **P value** |
| β2 microglobulin | 0.419 | 0.033 |
| BTLA | -0.466 | 0.014 |
| CCR10 | -0.432 | 0.022 |
| CD11a | -0.398 | 0.033 |
| CD134 | 0.475 | 0.009 |
| ADAM10 | -0.449 | 0.015 |
| CD196 | -0.403 | 0.030 |
| CD200R | -0.421 | 0.023 |
| CD22 | -0.515 | 0.004 |
| CD231 | -0.533 | 0.003 |
| CD245 | -0.454 | 0.013 |
| CD270 | -0.386 | 0.047 |
| CD275 | -0.368 | 0.050 |
| CD307d | -0.380 | 0.042 |
| CD314 | -0.439 | 0.017 |
| CD338 | -0.442 | 0.018 |
| CD35 | -0.391 | 0.036 |
| CD352 | -0.424 | 0.025 |
| CD39 | -0.425 | 0.024 |
| CD45 | -0.382 | 0.041 |
| CD47 | -0.506 | 0.005 |
| CD48 | -0.424 | 0.025 |
| CD49d | -0.417 | 0.027 |
| CD50 | -0.478 | 0.009 |
| CD52 | -0.371 | 0.048 |
| CD53 | -0.408 | 0.028 |
| CD55 | -0.484 | 0.009 |
| CD71 | -0.516 | 0.004 |
| CD81 | -0.390 | 0.036 |
| HLA-DQ | -0.449 | 0.017 |
| HLA-E | -0.377 | 0.048 |
| NKp80 | -0.432 | 0.019 |
| Notch 1 | -0.382 | 0.045 |

**
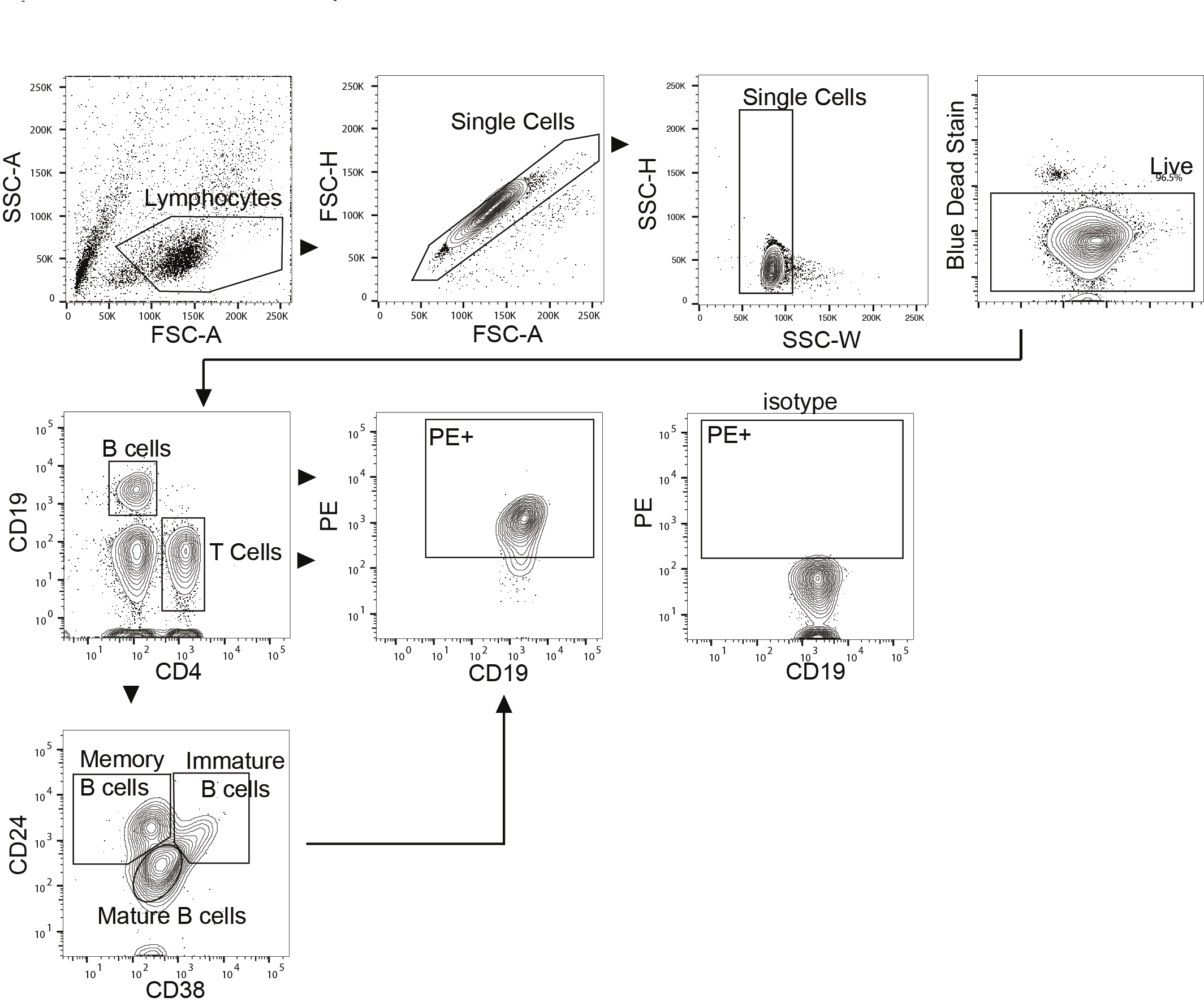
Supplementary Figure 1. LEGENDScreen^TM^ gating strategy.** Representative flow cytometry plots showing the sequential gating strategy for CD19^+^B cells, CD4^+^T cells and B cell subsets based on CD24 and CD38 expression on CD19^+^B cells.

**
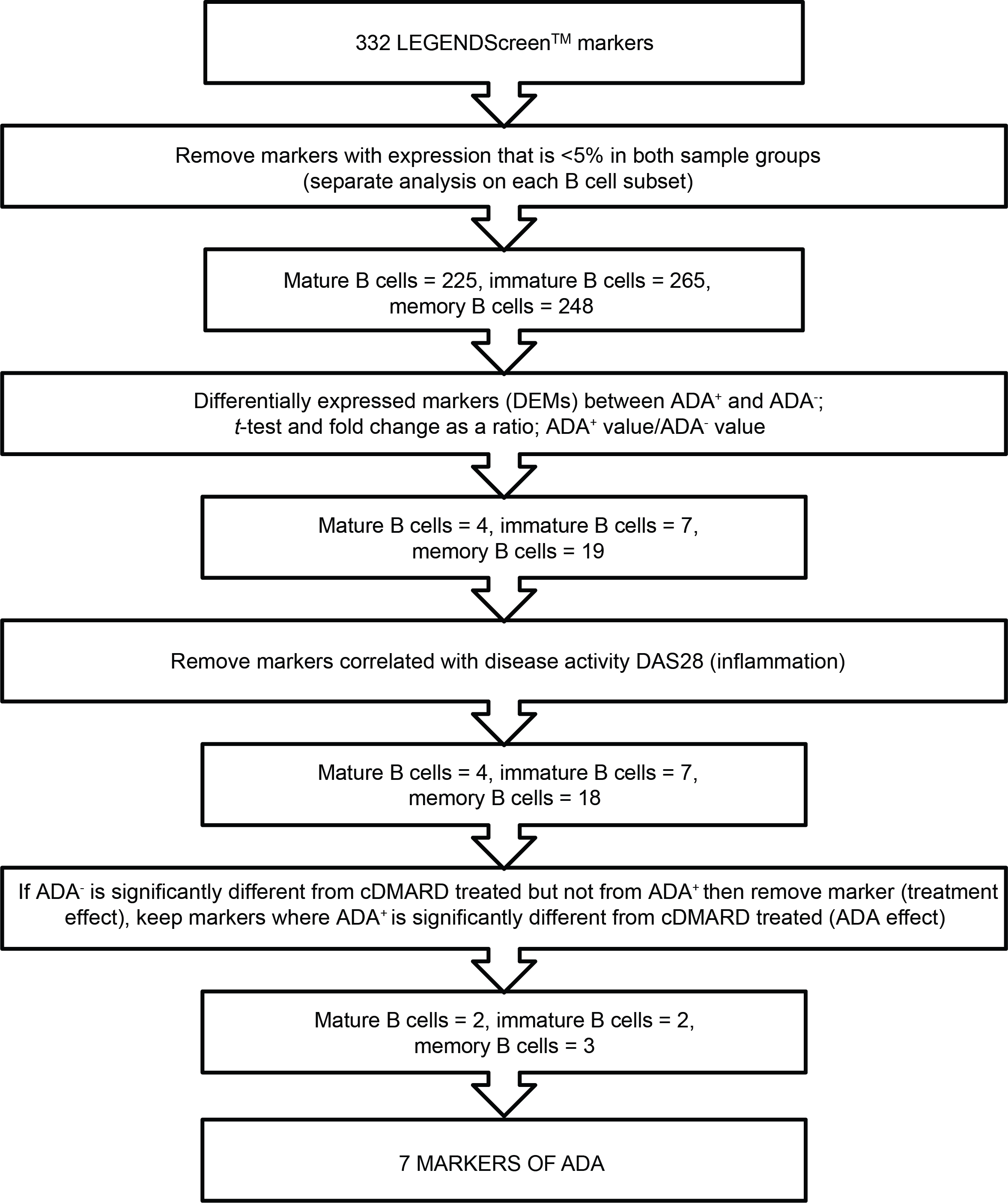
**

**Supplementary Figure 2. Selection of DEMs for ADA module** **from LEGENDScreen^TM^ analysis of cross-sectional cohort.** (A) Flow Diagram. One-way ANOVA analysis of ADA^-^ vs. ADA^+^ vs. cDMARD treated (RA-D) RA patients (see also Figure S3). Fold change calculated as a ratio of the ADA^+^ value divided by the ADA^-^ value.

**
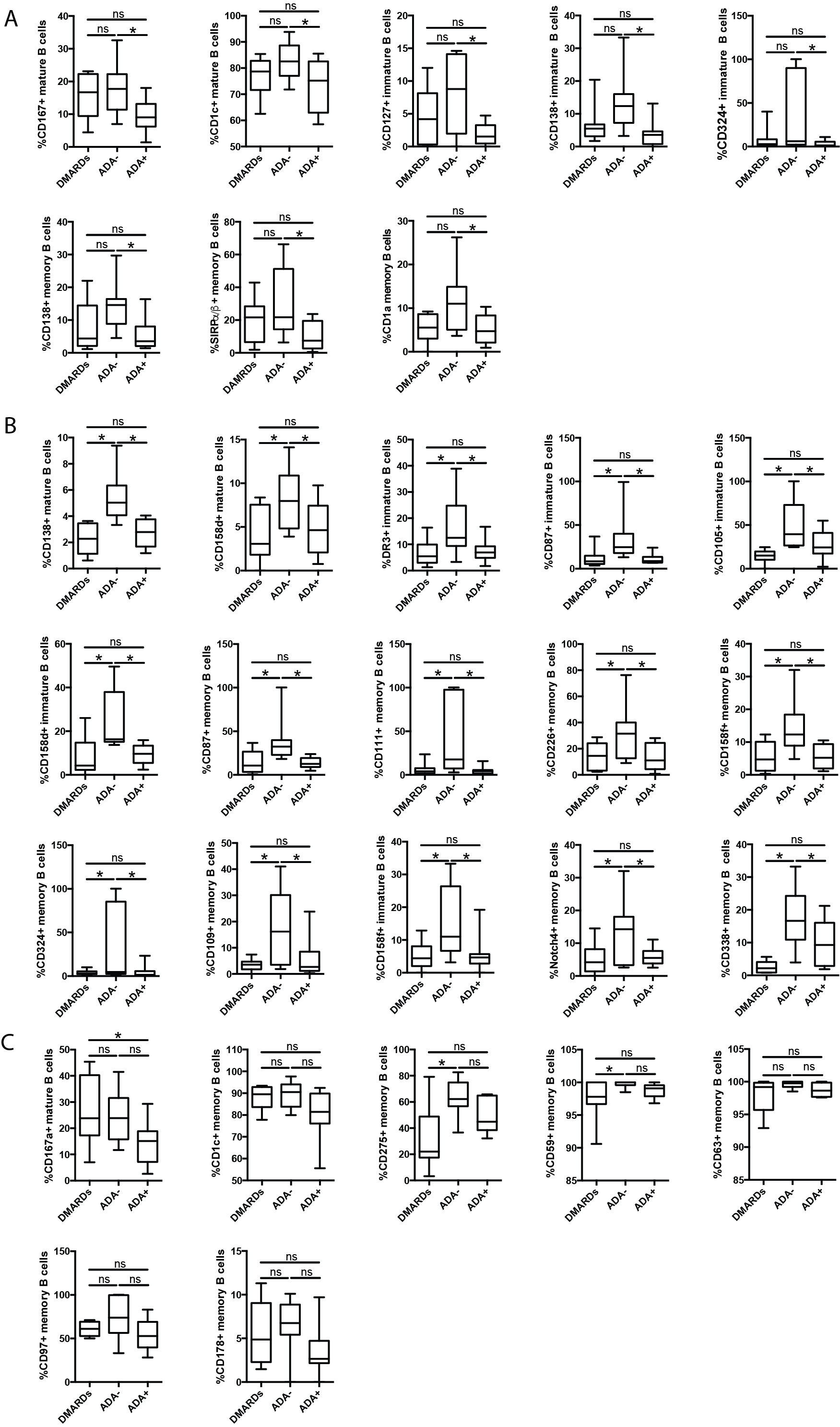
Supplementary Figure 3. Removal of DEMs from ADA module.** One-way ANOVA analysis of ADA^-^ vs. ADA^+^ vs. RA-D RA patients; (A) markers that were retained, (B) markers removed due to significant difference between ADA^-^ and RA-D, and (C) markers removed as ADA^-^ vs. ADA^+^ no longer significant. Box and whisker plot (min to max) *p≤0.05, ns=not significant.

**
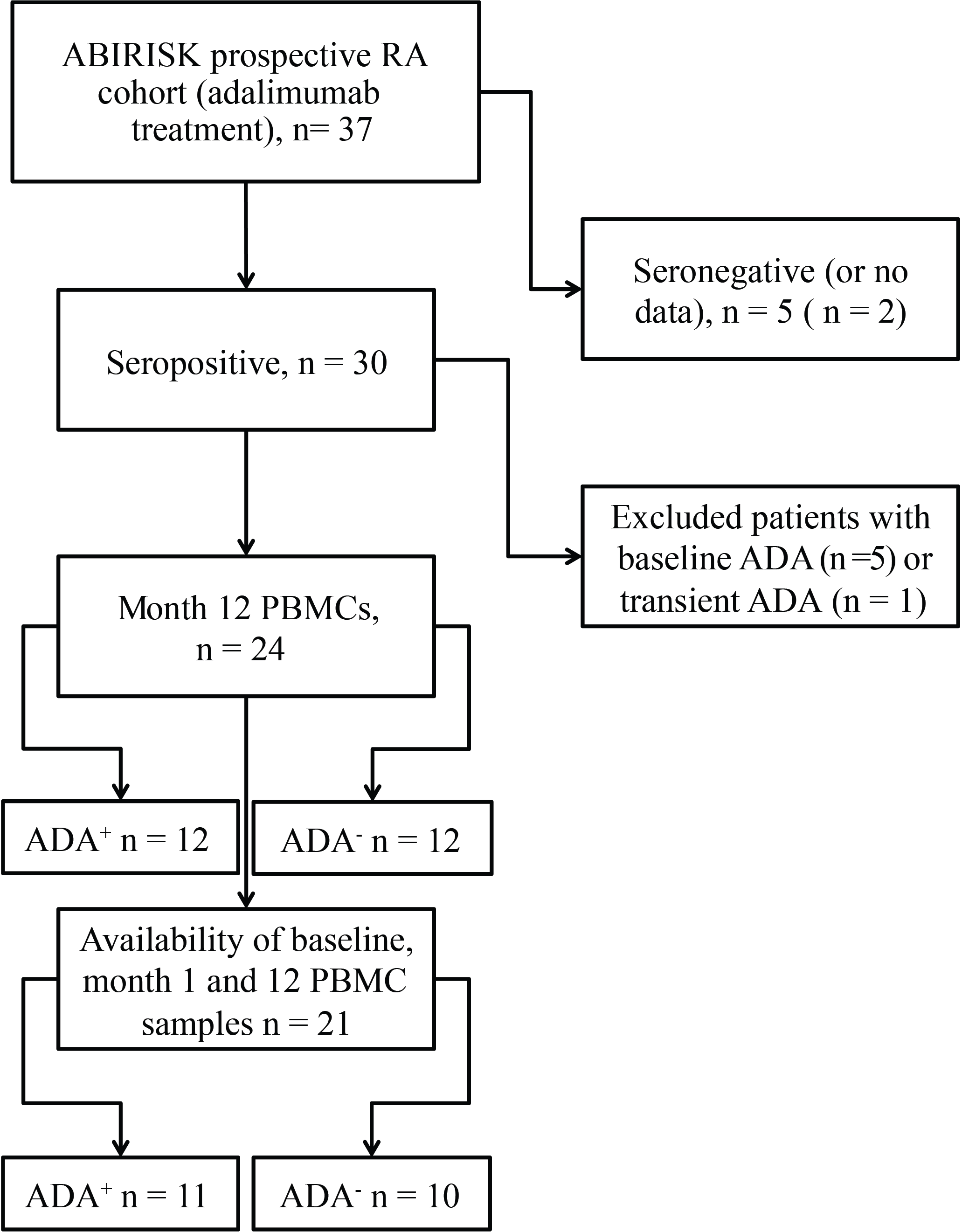
**

**Supplementary Figure 4. Selection of prospective patients for validation cohort.** Flow Diagram detailing the selection criteria applied to the validation cohort of patients.

**
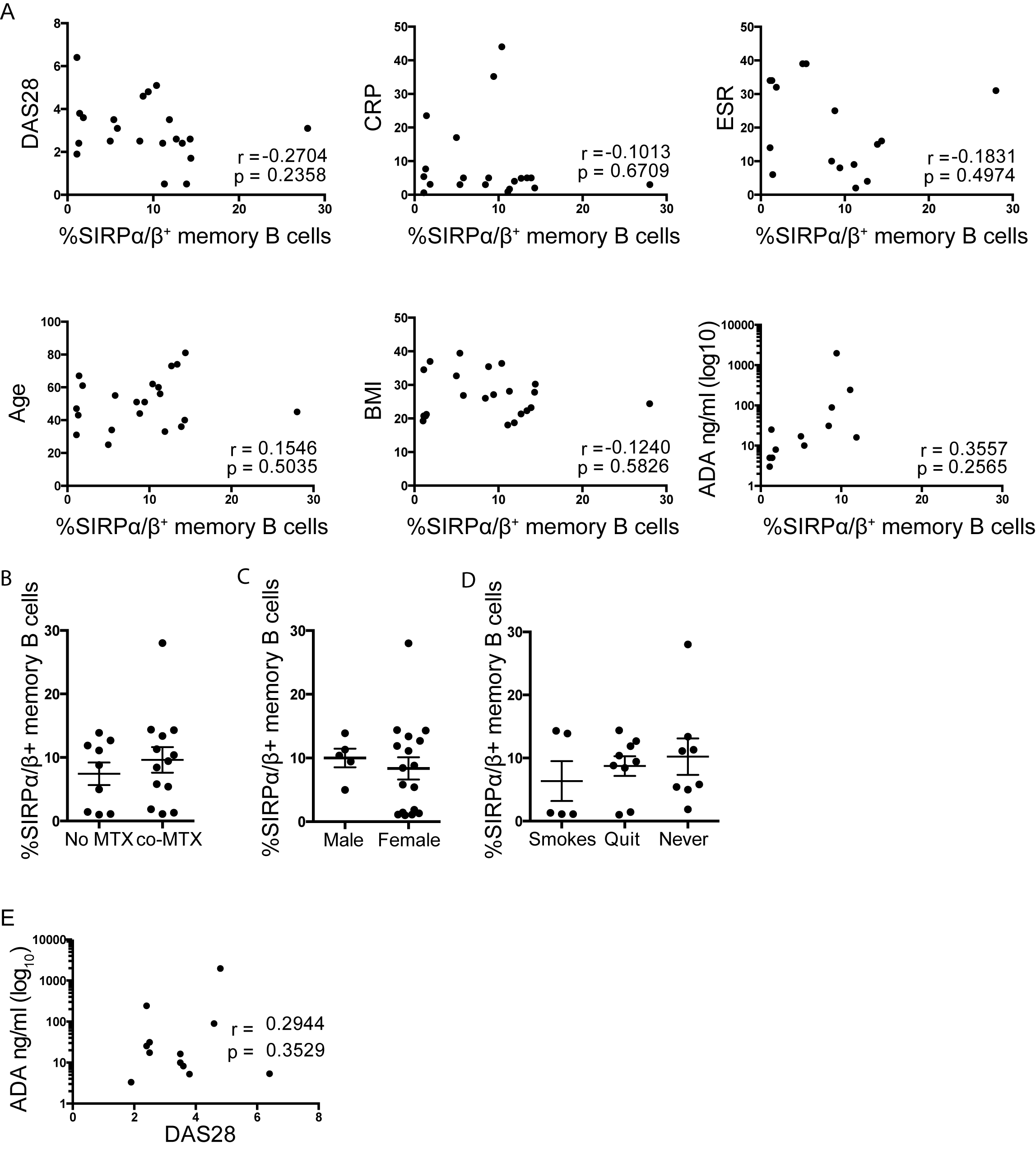
**

**Supplementary Figure 5. SIRPα/β, ADA, and clinical data.** Clinical data for prospective cohort patients was recorded. (A) %SIRPα/β^+^memory B cells with: DAS28 score, CRP mg/L, ESR mm/hr, age, BMI, and ADA ng/ml (log_10_), at month 12. %SIRPα/β^+^memory B cells (B) in patients treated with combination therapy with methotrexate (co-MTX), or without (No MTX) (C) gender and (D) smoking status, at month 12. (E) ADA ng/ml (log_10_) and DAS28. All non significant (p>0.05). For A and E Pearson Correlation (r) was performed, and for B and C *t*-test and D one-way ANOVA.

**Supplementary Figure 6. Frequency of SIRPα/β^+^ memory B cells in cDMARD treated RA patients classified as responders and non-responders.** Patients treated with cDMARDs only were stained for SIRPα/β^+^ on memory B cells and classified as responders or non-responders according to the EULAR classification. Dot plot ±SEM, not significant (p>0.05, *t*-test).
